# Liver autotransplantation and atrial reconstruction on a patient with multiorgan alveolar echinococcosis: a case report

**DOI:** 10.1186/s12879-024-09545-0

**Published:** 2024-07-02

**Authors:** Rexiati Ruze, Tiemin Jiang, Weimin Zhang, Mingming Zhang, Ruiqing Zhang, Qiang Guo, Aboduhaiwaier Aboduhelili, Musitapa Zhayier, Ahmad Mahmood, Zhaoxia Yu, Jianrong Ye, Yingmei Shao, Tuerganaili Aji

**Affiliations:** 1 Department of Hepatobiliary and Echinococcosis Surgery, Digestive and Vascular Surgery Center, The First Affiliated Hospital, Xinjiang Medical University, Urumqi, 830011 China; 2 State Key Laboratory of Pathogenesis, Prevention and Treatment of High Incidence Diseases in Central Asia, Xinjiang Medical University, Urumqi, 830011 China; 3 Department of Cardiac Surgery, The First Affiliated Hospital, Xinjiang Medical University, Urumqi, 830011 China; 4 Department of Critical Care Medicine, The First Affiliated Hospital, Xinjiang Medical University, Urumqi, 830011 China; 5 Department of Anesthesia, The First Affiliated Hospital, Xinjiang Medical University, Urumqi, 830011 China

**Keywords:** Hepatic alveolar echinococcosis, Cardiac alveolar echinococcosis, Liver autotransplantation, Atrial reconstruction, Case report

## Abstract

**Background:**

Alveolar echinococcosis (AE) primarily affects the liver and potentially spreads to other organs. Managing recurrent AE poses significant challenges, especially when it involves critical structures and multiple major organs.

**Case presentation:**

We present a case of a 59-year-old female with recurrent AE affecting the liver, heart, and lungs following two previous hepatectomies, the hepatic lesions persisted, adhering to major veins, and imaging revealed additional diaphragmatic, cardiac, and pulmonary involvement. The ex vivo liver resection and autotransplantation (ELRA), first in human combined with right atrium (RA) reconstruction were performed utilizing cardiopulmonary bypass, and repairs of the pericardium and diaphragm. This approach aimed to offer a potentially curative solution for lesions previously considered inoperable without requiring a donor organ or immunosuppressants. The patient encountered multiple serious complications, including atrial fibrillation, deteriorated liver function, severe pulmonary infection, respiratory failure, and acute kidney injury (AKI). These complications necessitated intensive intraoperative and postoperative care, emphasizing the need for a comprehensive management strategy in such complicated high-risk surgeries.

**Conclusions:**

The multidisciplinary collaboration in this case proved effective and yielded significant therapeutic outcomes for a rare case of advanced hepatic, cardiac, and pulmonary AE. The combined approach of ELRA and RA reconstruction under extracorporeal circulation demonstrated distinct advantages of ELRA in treating complex HAE. Meanwhile, assessing diaphragm function during the perioperative period, especially in patients at high risk of developing pulmonary complications and undergoing diaphragmectomy is vital to promote optimal postoperative recovery. For multi-resistant infection, it is imperative to take all possible measures to mitigate the risk of AKI if vancomycin administration is deemed necessary.

**Supplementary Information:**

The online version contains supplementary material available at 10.1186/s12879-024-09545-0.

## Background

Alveolar echinococcosis (AE) is a lethal parasitic infection caused by *Echinococcus multilocularis* that predominantly involves the liver [1]. Early diagnosis of hepatic AE (HAE) remains challenging due to long asymptomatic periods [2]. While initially benign, HAE invades surrounding tissues and can metastasize hematogenously to distant organs in approximately 3% of cases [1, 3]. Without treatment, the prognosis of HAE is poor, with metastasis rates exceeding 20% and 10-year mortality approaching 90% [3, 4]. Optimal management consists of radical surgical resection and chemotherapy, but these are only applicable to early-stage diseases [5]. For advanced HAE, liver transplantation currently offers the sole curative option [6], but faces limitations including donor availability, immunosuppression, financial cost, and high residual/recurrent rates [7].

In comparison, ex vivo liver resection and autotransplantation (ELRA) circumvents the challenges of liver transplantation while avoiding immunosuppression and providing comparable or superior outcomes [7]. While the efficacy of ELRA has been demonstrated for end-stage HAE, its application and outcomes in the rare setting of cardiac involvement remain poorly characterized. Here we present a rare case of multisystem AE involving the liver, heart, and lungs managed with complicated ELRA combined with right atrial (RA) reconstruction and pericardial/diaphragmatic repair. Through detailed description of the procedure and clinical course, this report aims to enhance our understanding of ELRA's role alongside cardiac surgery for advanced HAE complicated by cardiac invasion.

## Case presentation

### Medical history

The patient was a case of a 59-year-old Tibetan female from a known endemic region in Southwestern China who initially presented over a decade prior with persistent epigastric pain radiating to the scapula. She had been diagnosed with HAE ten years prior and underwent two hepatectomies as well as albendazole therapy. Despite initial symptom relief, her condition recurred and worsened with extrahepatic spread now involving the heart. Her past medical history was notable for poorly controlled hypertension and other comorbidities were denied.

### Preoperative assessments

**Fig. 1:**
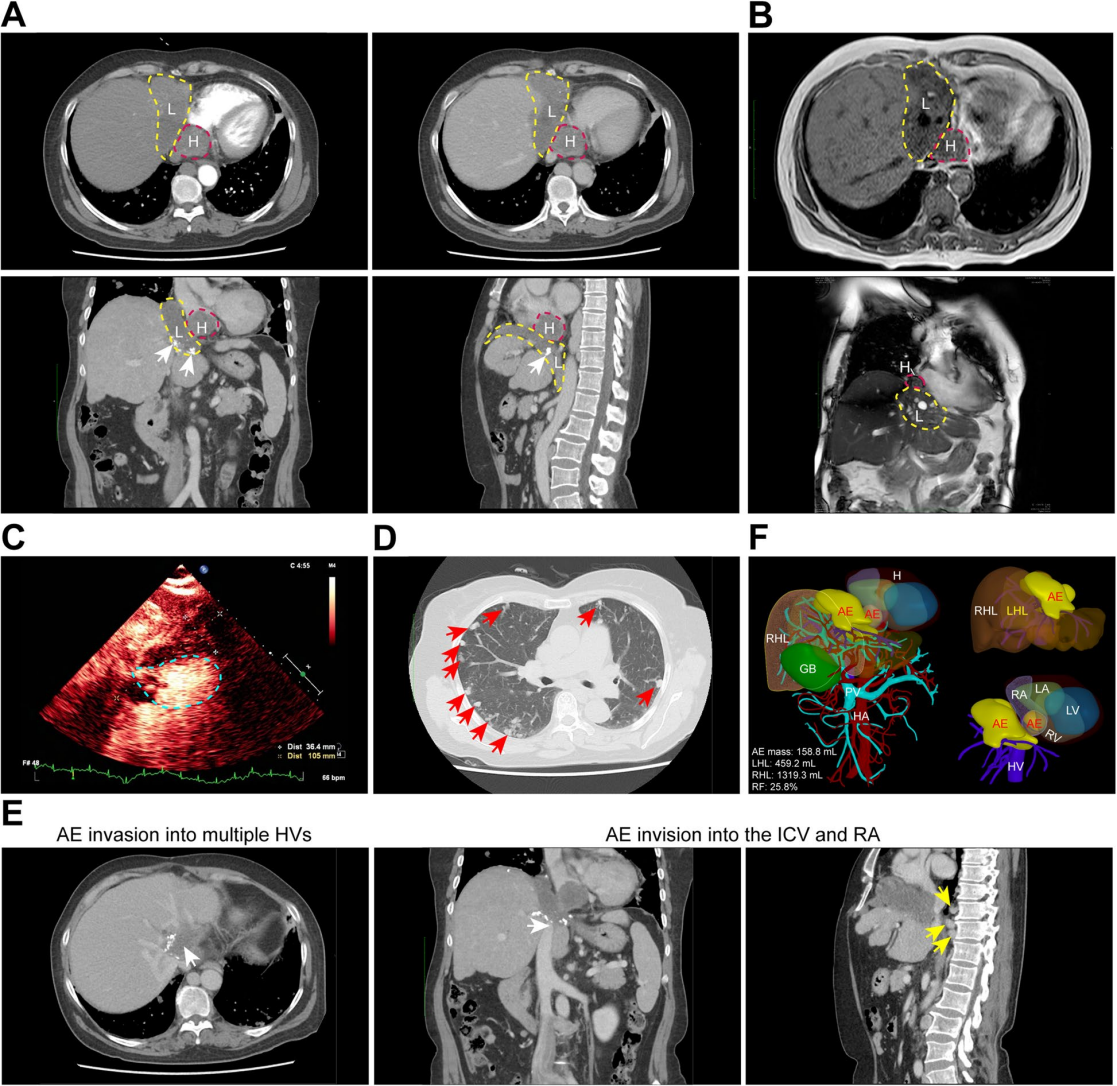
Preoperative imaging of the AE masses, indications for ex vivo liver resection and autotransplantation (ELRA), and volumetry of the patient’s liver. **A** Mixed density AE masses, delineated by yellow (L, liver) and red (H, heart) dashed circles, exhibit approximate dimensions of 7.11 × 6.02 cm, as discerned from the computed tomography (CT) and CT angiography (CTA). Situated around the right diaphragm, retrohepatic inferior vena cava (IVC), and the right atrium (RA), these unenhanced masses during the arterial phase (upper left) also displayed scattered calcification foci (white arrows; venous phase: upper right; coronal plane: bottom left; sagittal plane: bottom right). The venous phase further uncovered a mild dilation of the right branch of the portal vein (PV), disarray of the second hepatic portal, and an indistinct representation of the left hepatic vein (HV), and compression and narrowing of the retrohepatic IVC, attributed to the pressure from the masses. **B** The abdominal (above) and cardiac (below) magnetic resonance imaging (MRI) scans. The AE lesions, marked by yellow (L, liver) and red (H, heart) dashed circles, were identifiable in the left lobe, caudate lobe, and tail lobe of the liver. With indistinct boundaries, these lesions measured approximately 7.96 × 3.45 cm at their largest extent. The left branch of the PV exhibited partial obscurity, and the HVs are thickened (below); concurrently, a mass was discerned near the RA. **C** A cystic-solid mixed mass observed in the cardiac ultrasound angiography, located below the diaphragm near the RA side wall at the hepatic portal, and this mass was suspectedly located within the RA. **D** CT angiography (CTA) shows symmetrical thorax with diffused nodules and blocky lesions in both lungs (indicated by red arrows). The largest measured about 1.86 × 1.60 cm. Slight atelectasis was noted in the right middle lobe's pericardial outer edge and the left upper lobe's lingual segment. Multiple mediastinal and hilar lymph nodes with increased density suggest homologous pulmonary AE lesions. **E** Abdominal CT scans indicate a severe invasion of the AE lesion into multiple HVs, IVC, and the RA (white arrows), with numerous circulating lateral branches forming around the blood vessels of the vertebrae (yellow arrows). **F** The three-dimensional (3D) reconstruction of the patient’s liver, heart, AE lesions, and major vessels, along with the volumetry of the liver and the lesions (Available at: https://yun.gradient3d.com/scene/case3d.html?ValidCode=230709&SID=b94e05f8-4e36-4773-8c3c-29fa31696896). The residual liver post-resection (right hemiliver, RHL) is highlighted in yellow. AE, alveolar echinococcosis; GB, gall bladder; H, heart; HA, hepatic artery; HV, hepatic vein; LA, left atrium; LHL, left hemiliver; LV, left ventricle; PV, portal vein; RA, right atrium; RF, resection fraction; RHL, right hemiliver; RV, right ventricle. For comprehensive images of the CT and MRI scans, kindly refer to the accompanying videos

The patient presented with jaundice, abdominal discomfort, proteinuria and elevated blood glucose (Supplementary Table 1). Ultrasonic imaging revealed an irregular hepatic lesion measuring approximately 8.0 × 3.0 × 6.2 cm involving the liver lobes with unclear boundaries (Supplementary Fig. 1A). Computed tomography (CT) identified mixed density masses in the liver and heart measuring 7.11 × 6.02 cm near the diaphragm, retrohepatic inferior vena cava (IVC) and right atrium (RA), exhibiting scattered calcification and compression of surrounding structures (Fig. 1A). Magnetic resonance imaging (MRI) showed the lesions resulted in partial obscuration of the left portal vein (PV) branch and thickening of the hepatic veins (HVs) (Fig. 1B and Supplementary Fig. 1B). Echocardiography revealed a mass without blood flow at the junction of the RA and IVC, increasing IVC flow velocity (Supplementary Fig. 1C). Ultrasonic cardiac angiography identified a cystic-solid intra-atrial mass within the RA, which was later confirmed on transesophageal echocardiography (TEE) (Fig. 1C and Supplementary Video 1).

Pulmonary function tests indicated type 1 respiratory failure and moderate mixed ventilatory dysfunction, plus increased peripheral elastic resistance (Supplementary Table 2). Computed tomography angiography (CTA) further identified atelectasis in the middle lobe of the right lung and the upper lobe of the left lung, accompanied by diffuse nodular and blocky lesions in both lungs, indicative of coexisting pulmonary AE (PAE) (Fig. 1D). Based on preoperative imaging, the patient was determined to be at stage P_4_N_1_M_1_, signifying definitive end-stage AE according to the previous World Health Organization (WHO) classification [8]. Consequently, a multidisciplinary strategy was proposed involving radical excision of the hepatic and cardiac lesions, supplemented with postoperative oral albendazole therapy to manage the PAE.

### Surgical procedures

#### Surgical plan

Preoperative imaging demonstrated extensive invasion of recurrent AE lesions into the second porta hepatis, multiple hepatic veins (HVs), and majority of the right atrium. Significant inferior vena cava narrowing from extrahepatic invasion led to collateral circulation (Fig. 1E). These conditions necessitated radical lesion resection and complex reconstruction of the right atrium and inferior vena cava. However, high bleeding risk and challenge of complete resection rendered in vivo procedure problematic. Therefore, an extended right hepatic lobectomy, RA reconstruction, pericardial and diaphragmatic repair was proposed following a multidisciplinary team (MDT) discussion. The estimated residual volume of the right hepatic lobe was 1319.3 mL, correlating with a resection fraction of 25.8% and a remnant liver volume of 105.6% (Fig. 1F and Supplementary Fig. 1D). This estimation indicated significant hypertrophy of the patient’s healthy liver lobe due to compensatory mechanisms, yet affirming both the feasibility and safety of the proposed surgical approach [9]. Owing to the high position of CAE and its encroachment into the RA, the clamping and resection of the affected atrial region would impede the return flow of cardiac blood. Consequently, the Dpts. Cardiac Surgery and Anesthesiology collectively advocate for the resection of the CAE lesion under the facilitation of extracorporeal circulation.

#### In vivo liver dissection, extracorporeal circulation establishment, and en bloc resection

**Fig. 2:**
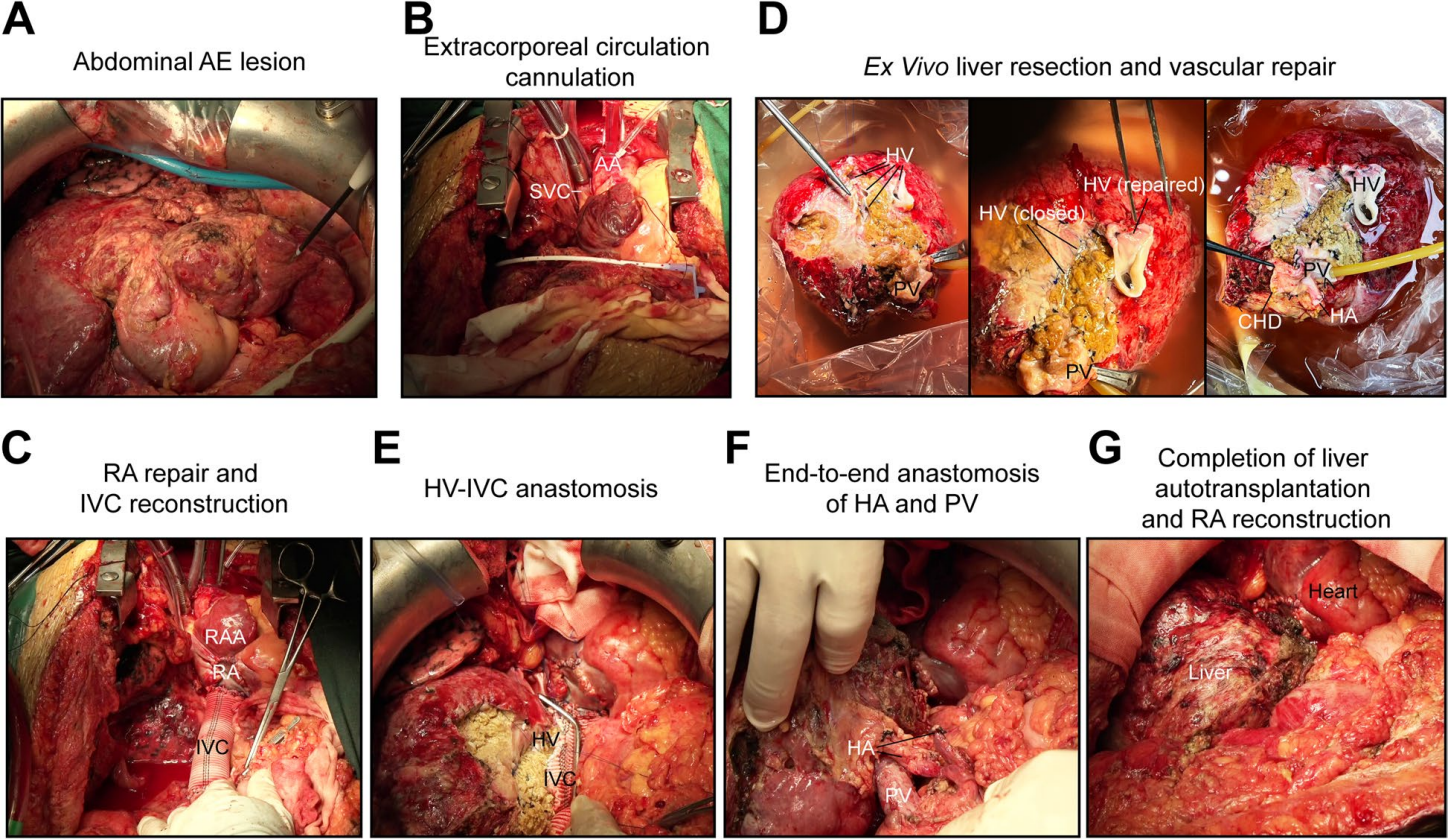
Main steps of the surgical procedures. **A**-**G** The fundamental steps of ELRA and RA reconstruction. AA, ascending aorta; AE, alveolar echinococcosis; CHD, common hepatic duct; HA, hepatic artery; HV, hepatic vein; IVC, inferior vena cava; PV, portal vein; RA, right atrium; RAA, right atrial appendage; SVC, superior vena cava. For a comprehensive understanding of the surgical procedures, please refer to the uploaded videos

The patient underwent a laparotomy in the supine position under general anesthesia. Due to two previous hepatectomies, the liver structure was disordered and densely adhered (Fig. 2A). AE had infiltrated the middle and right HVs, bile ducts, retrohepatic IVC. Following a dissection of the primary hepatic portal (Supplementary Fig. 2A), the HA, PV, and bile ducts, a separation of the retrohepatic IVC along the posterior peritoneum, a diaphragmectomy was performed to remove lesions and expose pericardial and atrial invasion. Due to the high cardiac lesion position and challenge occluding right atrial reflux, a thoracotomy and cardiopulmonary bypass were utilized (Fig. 2B and Supplementary Fig. 2B). An *en* bloc resection included the entire liver, retrohepatic IVC, bilateral diaphragm, pericardium, and RA (Supplementary Fig. 2C). The excised liver was promptly perfused with histidine-tryptophan-ketoglutarate solution (CUSTODIOL, 2000 mL) at 0–4 °C for ex vivo resection.

#### RA repair, IVC reconstruction, and ex vivo liver resection

RA repair was performed using extracorporeal circulation with a heart–lung machine (Jostra, H2O) and oxygenator (TERUMO, CX*RX25RW). Following systemic heparinization, ACT was maintained at 824 s. The RA was reconstructed with a surgical patch (Guanhao Biotech, TB-S80120) containing an opening for IVC anastomosis. An artificial blood vessel (GORE-TEX, Stretch Vascular Graft, SA2002) was sutured to the IVC opening. Upon completion, end-to-end anastomoses were established between the autologous IVC and reconstructed IVC (Fig. 2C and Supplementary Fig. 2E). After heparin neutralization, extracorporeal circulation was discontinued (Supplementary Fig. 2F). Meanwhile, the AE lesions were meticulously excised from the diseased liver along boundaries using a cavitron ultrasonic surgical aspirator (CUSA) (Söring, SONOCA 300). During this, the left HV, HA, PV and caudate lobe were entirely excised. The right HVs were reconfigured to facilitate subsequent anastomosis with the IVC (Fig. 2D). Finally, the prepared liver weighed 1200 g.

#### Liver autotransplantation

Prior to autotransplantation, the patient received a plasma transfusion to optimize coagulation. The resected liver was positioned and the right HV was anastomosed to the synthetic IVC (Fig. 2E and Supplementary Fig. 2G), followed by an in situ anastomosis of the repaired PV (Fig. 2F and Supplementary Fig. 2H). Restoration of blood flow was achieved by opening the PV. Subsequently, the trimmed right branch of the proper hepatic artery (HA) was anastomosed with its main trunk (Fig. 2F and Supplementary Fig. 2I), followed by anastomosis of the common hepatic duct (CHD) to the common bile duct (CBD) (Fig. 2G). The pericardium and diaphragm were repaired using the surgical patches (Guanhao Biotech, TB-S80120). Intraoperative ultrasound verified adequate blood supply of the transplanted liver. Total blood loss was 2800 mL with intravenous transfusions including crystalloids, colloids, autologous blood, red blood cells and plasma. The 17 h 40 min procedure featured a 3-h 45 min anhepatic phase. The patient was then moved to the intensive care unit (ICU) (Supplementary Table 2).

#### Postoperative management

**Fig. 3:**
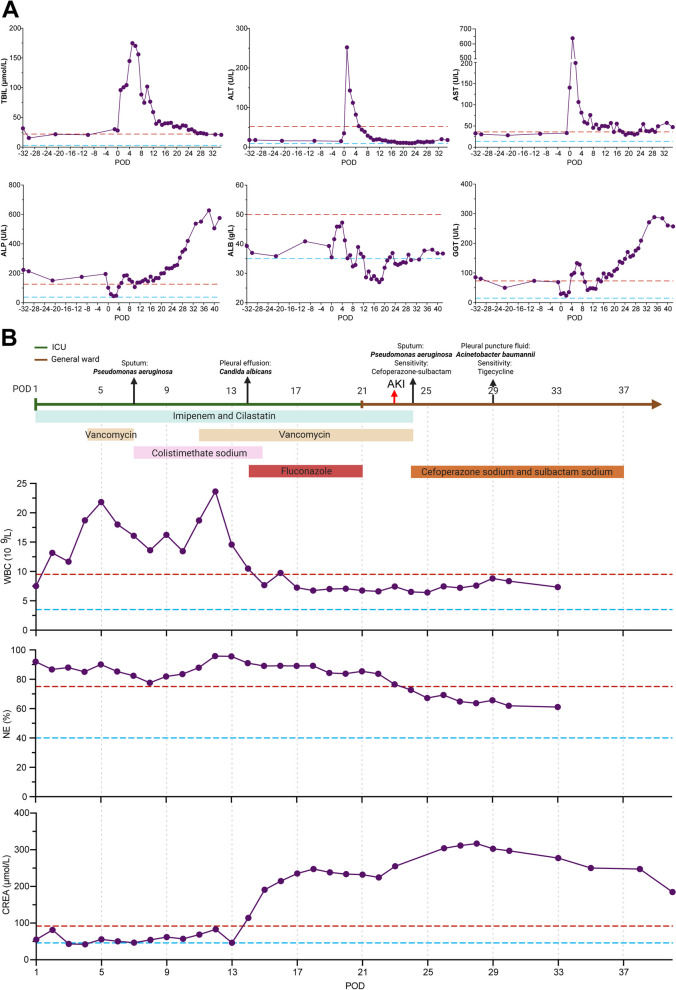
Perioperative monitoring of the liver function and anti-biotic regimens for pulmonary infection and subsequent vancomycin-associated acute kidney injury. **A** Various parameters of liver function. The upper and lower boundaries of normality are respectively denoted by dashed red and blue lines. **B** Different anti-infections regimens during the postoperative period. The different colors on the horizontal arrow indicate the ward of the patient. Vertical arrows denote the timing of microbiological culture results and AKI diagnosis. Rectangles of different colors represent distinct anti-infective agents, demonstrating their durations. The effects of these drugs on infection control and kidney function are shown in the line charts below. AKI, acute kidney injury; ALB, albumin; ALP, alkaline phosphatase; ALT, alanine aminotransferase; AST, aspartate aminotransferase; CREA, creatine; GGT, gamma-glutamyltransferase; HGB, hemoglobin; ICU, intensive care unit; NE, neutrophils; POD, postoperative day; TBIL, total bilirubin; WBC, white blood cell

With a vulnerable cardiopulmonary function, the patient underwent extensive postoperative management for an APACHE II score [10] of 14. Her treatment regimen incorporated antipyretics, oxygen therapy, repeated bronchoalveolar lavage (BAL) for sputum clearance, and a multidrug antibiotic protocol. Postoperatively, point-of-care ultrasound (POCU) on postoperative day (POD) 1 revealed minimal perihepatic hematoma (PHH) and pericardial effusion. However, the patient developed atrial fibrillation on POD2, which was successfully cardioverted with defibrillation via the pacemaker lead implanted on the surface of the right ventricle during surgery. Repeated BAL procedures failed to adequately clear sputum and support lung function, resulting in persistent pulmonary infection. From POD5, adenosine and glycyrrhizin injections were administered to manage worsening liver function (Fig. 3A). Sputum cultures on POD7 grew *Pseudomonas aeruginosa*, necessitating the addition of colistin (Fig. 3B) and increased frequency of BAL. A tracheostomy was later performed on POD 11 due to pericardial effusion and bilateral pneumonia identified on CT. On POD14, fluconazole was initiated to treat multidrug-resistant *Candida albicans* fungemia. The patient was transferred back to the general ward on POD21 after attaining infection control. However, vancomycin-associated acute kidney injury (VA-AKI) necessitated regimen adjustment and preventative heart failure maneuvers (Fig. 3B and Supplementary Fig. 3A). Afterwards, POCU demonstrated left calf intermuscular vein thrombosis, obligating further antithrombotic therapy, monitoring of coagulation only showed a slight increase in fibrinogen (Supplementary Fig. 3B). Subsequently, the patient gradually recovered, but with hypohemoglobinemia, hypokalemia, and hyponatremia (Supplementary Fig. 3C and 3D). Considering that the severe pulmonary infection was solved, and the patient’s condition was gradually improved, she was discharged on POD 42 with prescriptions for analgesics and anticoagulant medications, dietary guidance, alongside comprehensive PAE treatment and follow-up recommendations. A comprehensive record of the diagnostic and treatment trajectory can be found in Supplementary Table 2.

**Fig. 4:**
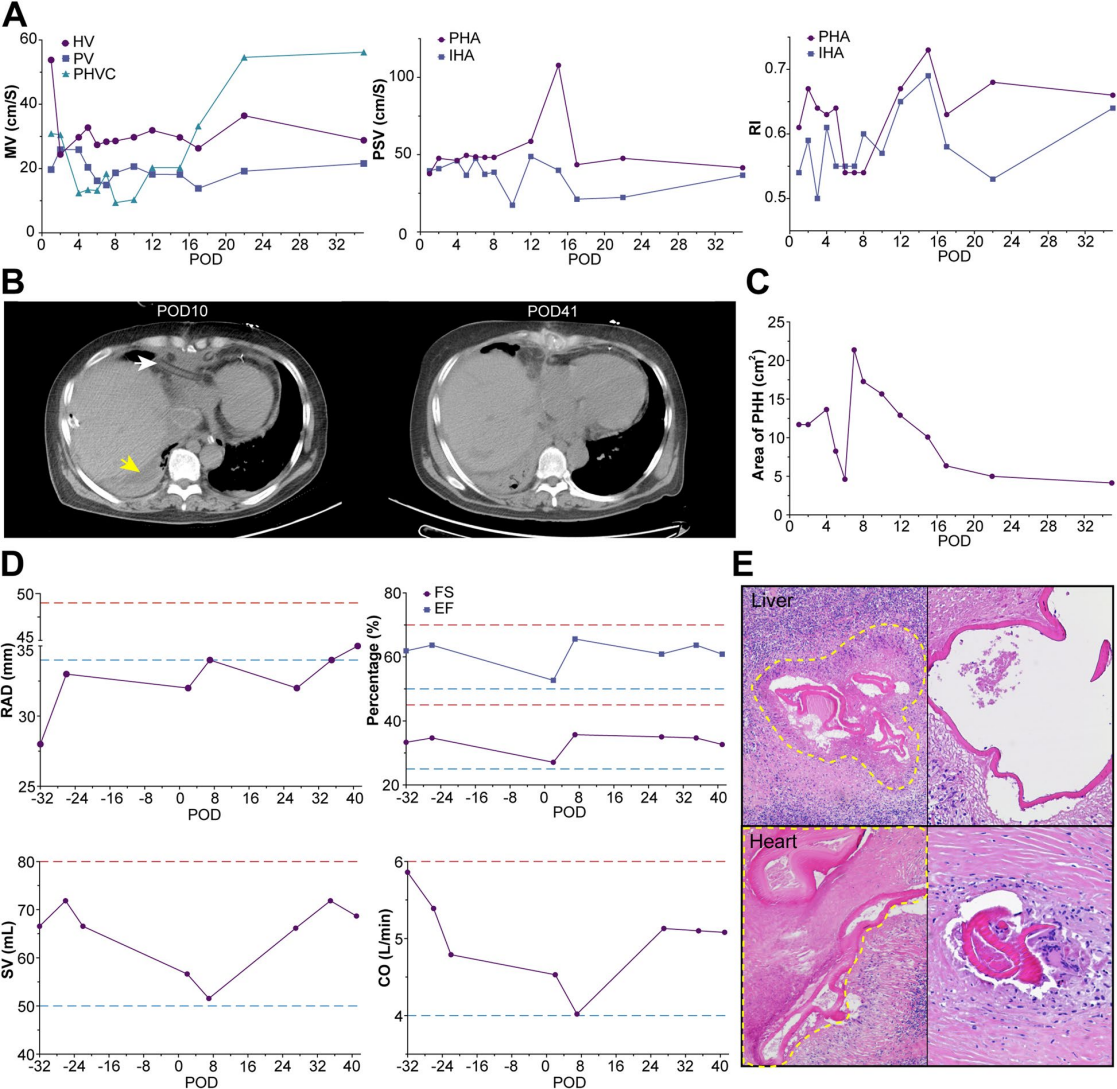
Postoperative assessment of blood flow of transplanted liver, recovery and cardiac function of the patient, and pathology of excised lesions. **A** The mean velocity (MV), peak systolic velocity (PSV), and resistance index (RI) of the HV, PV, retrohepatic vena cava (PHVC), portal hepatic artery (PHA), and intrahepatic artery (IHA) recorded on successive postoperative days (POD). **B** Postoperative abdominal CT scans, revealing the artificial IVC, pericardial drainage tube (indicated by the white arrow), and perihepatic hematoma (PHH, marked by the yellow arrow) at POD10. By POD41, the tube had been removed, and the PHH was no longer visible. **C** The areas of PHH as measured via abdominal ultrasound across different PODs. **D** The right atrium diameter (RAD) and cardiac function measured under echocardiography post RA reconstruction, with the red and blue dashed lines signifying the highest and lowest normal values, respectively. Key metrics include cardiac output (CO), ejection fraction (EF), fractional shortening (FS), and stroke volume (SV). **E** Representative pathological images of the HAE and CAE, the lesions are indicated by yellow dashed circle. Left, × 100; right, × 400. CAE, cardiac alveolar echinococcosis; HAE, hepatic alveolar echinococcosis; HV, hepatic vein; IHA, intrahepatic artery; PHVC, post hepatic vena cava; PV, portal vein; PHA, portal hepatic artery; S, second

Postoperative monitoring of the patient involved multiple modalities to assess organ function following ELRA. POCU was used serially to evaluate blood flow in the transplanted liver, demonstrating progressive enhancement and subsequent stabilization of hepatic function over time (Fig. 4A). Concurrently, CT scans and POCU showed a notable reduction in pleural effusions and PHH following the procedure (Fig. 4B and C). ECG after RA reconstruction indicated restoration of normal RA diameter and satisfactory cardiac performance (Fig. 4D). Histopathological examination of biopsy specimens from the liver and heart lesions observed preoperatively confirmed the diagnosis of AE, consistent with clinical findings (Fig. 4E).

#### Follow-up and outcomes

Follow-up assessments performed at three months post-discharge involved clinical examination, blood tests, and chest/abdominal CT scans at the local hospital, which showed the patient's blood tests were normal. CT scans demonstrated the PAE was well-controlled with albendazole, showing only minimal peritoneal fluid around the liver with no abnormalities or signs of AE recurrence.

## Discussion and conclusions

To the best of our knowledge, this is the first human case of ELRA combined with RA reconstruction under cardiopulmonary bypass. In this case of multiorgan AE, hepatic and cardiac AE were treated with surgery while PAE was managed with long-term albendazole therapy. This case highlights our extensive experience treating complex multiorgan AE, despite a hospital stay over 70 days and postoperative challenges. However, diaphragmatic repair's impact on lung function and increased infectious risk were perhaps underestimated given preexisting lung atelectasis. Subsequent prolonged anti-infection therapy indirectly led to prolonged vancomycin use and acute renal impairment, delaying recovery.

Metastatic spread of HAE has been well described for many organs [11]. In fact, extrahepatic AE is more difficult to diagnose due to its rare occurrence and variable symptoms [12]. Current, further exploration is needed to uncover metastatic features and organ tropism of HAE [4, 13]. Still, liver transplantation serves as an ultimate solution for advanced inoperable HAE [14], but it has higher mortality and recurrence versus resection alone [5]. By contrast, ELRA offers a novel approach for unresectable HAE, addressing limitations of allotransplantation by avoiding donor organs or immunosuppression [15].

CAE is a rare but serious manifestation typically resulting from direct extension of primary hepatic or pleural lesions, which requires a high index of clinical suspicion due to non-specific presentations including arrhythmia, myocardial infarction, and purulent pericarditis [16]. So far, only a few CAE cases have been reported and previous treatments ranged from simple cardiac surgery [17] to complex combined heart‐liver transplantation (CHLT) [18]. While initially successful, the patient underwent CHLT experienced multiple complications and infections, prolonging her hospitalization, and small pulmonary metastases also occurred due to immunosuppression.

Our patient faced high postoperative complication risk, particularly pulmonary infection, following hepatectomy due to diaphragm dysfunction [5, 19]. Previous liver transplant patients for end-stage HAE also experienced prolonged intensive care and tracheostomy due to ventilator dependence [20]. The patient's infection resulted from preoperative atelectasis and diffuse PAE, and pleural effusion/diaphragm dysfunction from diaphragmectomy [19]. Abdominal signs signal systemic infection, while surgical injury and compromised lungs potentially cause pleural effusion/pulmonary infection after ELRA [7, 19]. Approximately 10% of patients suffer from persistent postoperative diaphragm dysfunction after cardiac surgery [21, 22]. Factors like age, lifestyle, nutrition, phrenic nerve injury, ventilation, inflammation, and atelectasis primarily cause diaphragm dysfunction [23, 24]. Once it happens, persistent diaphragm dysfunction leads to prolonged ventilation and infections [22]. Instructive perioperative evaluation methods include several parameters of ultrasonography [21–23]. Preventive strategies for high-risk patients incorporate ventilation, surgical technique optimization, inspiratory muscle training, and early mobilization [21–23].

Besides, nearly one-quarter of patients treated with vancomycin will develop AKI [25]. Vancomycin-associated AKI (VA-AKI) is a significant complication after liver transplantation given recipient vulnerability to AKI and need for postoperative vancomycin [26]. Previous studies found imipenem-cilastatin increases VA-AKI risk [25, 27]. Conversely, imipenem-cilastatin/relebactam protects mice kidneys from vancomycin [28]. The creatine level of the patient began to rise since the fluconazole was added to the anti-infection regimen. Consistently, it was shown that colistin and fluconazole also increase VA-AKI risk [26, 29, 30]. Therefore, Vancomycin should be administered cautiously in critically ill patients, avoiding prolonged use/nephrotoxic combinations and maintaining troughs < 15.4 mg/L [30]. Alternative antibiotics, limited durations (< 2 weeks), and renal protection for high-risk patients (e.g. liver transplant recipients, diabetics, voriconazole users) are advised [26, 30, 31]. Continuous infusion is preferable to intermittent dosing [31]. In general, careful fluid/hemodynamic management, antibiotic monitoring, early renal replacement, and alternative therapies can help prevent or manage VA-AKI. Further research is needed to develop more advanced and automatic detection models base on algorisms to favor an early identification of VA-AKI.

The multidisciplinary collaboration in this case proved effective and yielded significant therapeutic outcomes for a rare case of advanced hepatic, cardiac, and pulmonary AE. The combined approach of ELRA and RA reconstruction under extracorporeal circulation demonstrated distinct advantages of ELRA in treating complex HAE. We also emphasize the importance of assessing diaphragm function during the perioperative period, especially in patients at high risk of developing pulmonary complications and undergoing diaphragmectomy, to promote optimal postoperative recovery. Last but not the least, it is imperative to take all possible measures to mitigate the risk of AKI if vancomycin administration is deemed necessary.

### Patient perspective

The patient and their family expressed heartfelt gratitude to all the medical staff, thanking them for their superb medical skills, professional ethics, and dedication in jointly performing such a complex major surgery, as well as for the care and assistance they provided during hospitalization.

### Supplementary Information

Supplementary Material 1.

## Data Availability

The data used to create the figures in this article are available from the corresponding authors upon reasonable request.

## Abbreviations

AE: Alveolar echinococcosis
AKI: Acute kidney injury
BAL: Bronchoalveolar lavage
CBD: Common hepatic duct
CHD: Common bile duct
CHLT: Combined heart‐liver transplantation
CT: Computed tomography
CTA: Computed tomography angiography
CUSA: Cavitron ultrasonic surgical aspirator
ELRA: *Ex vivo* Liver resection and autotransplantation
HA: Hepatic artery
HAE: Hepatic alveolar echinococcosis
HV: Hepatic vein
ICU: Intensive care unit
IVC: Inferior vena cava
MDT: Multidisciplinary team
MRI: Magnetic resonance imaging
PAE: Pulmonary alveolar echinococcosis
PHH: Perihepatic hematoma
POCU: Point-of-care ultrasound
POD: Postoperative day
PV: Portal vein
RA: Right atrium
TEE: Transesophageal echocardiography
VA-AKI: Vancomycin-associated acute kidney injury
WHO: World Health Organization
