# Correction: Liver autotransplantation and atrial reconstruction on a patient with multiorgan alveolar echinococcosis: a case report

**DOI:** 10.1186/s12879-024-09690-6

**Published:** 2024-08-06

**Authors:** Rexiati Ruze, Tiemin Jiang, Weimin Zhang, Mingming Zhang, Ruiqing Zhang, Qiang Guo, Aboduhaiwaier Aboduhelili, Musitapa Zhayier, Ahmad Mahmood, Zhaoxia Yu, Jianrong Ye, Yingmei Shao, Tuerganaili Aji

**Affiliations:** 1 Department of Hepatobiliary and Echinococcosis Surgery, Digestive and Vascular Surgery Center, The First Afliated Hospital, Xinjiang Medical University, Urumqi, 830011 China; 2 State Key Laboratory of Pathogenesis, Prevention and Treatment of High Incidence Diseases in Central Asia, Xinjiang Medical University, Urumqi, 830011 China; 3 Department of Cardiac Surgery, The First Afliated Hospital, Xinjiang Medical University, Urumqi, 830011 China; 4 Department of Critical Care Medicine, The First Afliated Hospital, Xinjiang Medical University, Urumqi, 830011 China; 5 Department of Anesthesia, The First Afliated Hospital, Xinjiang Medical University, Urumqi, 830011 China

Following publication of the original article [1], we have been notified that 3 documents were missing from the supplementary material .zip file:

- Supplementary materials.pdf.
- Surgical procedures.mp4.
- En bloc resection.mp4.

This has now been added to the linked .zip file.

The original article has been corrected.
