# Supplementary material for: Liver autotransplantation and atrial reconstruction on a patient with multiorgan alveolar echinococcosis: a case report
Source: BMC Infect Dis. 2024 Jul 2;24:659. doi: 10.1186/s12879-024-09545-0 (PMC11218102; doi:10.1186/s12879-024-09545-0)
Supplement: Supplementary file 1 — Supplementary Material 1. [file 12879_2024_9545_MOESM1_ESM.zip › 12879_2024_9545_MOESM1_ESM/Supplementary/Supplementary materials.pdf]

BMC Part of Springer Nature

BMC Infectious Diseases

SUPPLEMENTARY MATERIALS

Liver autotransplantation and atrial reconstruction on a patient with multiorgan alveolar echinococcosis: a case report

Rexiati Ruze[1,2†], Tiemin Jiang[1,2†], Weimin Zhang[3†], Mingming Zhang[3], Ruiqing Zhang[1], Qiang Guo[1], Aboduhaiwaier Aboduhelili[1], Musitapa Zhayier[1], Ahmad Mahmood[1], Zhaoxia Yu[4], Jianrong Ye[5], Yingmei Shao[1,2*], Tuerganaili Aji[1,2*]

[1]Department of Hepatobiliary and Echinococcosis Surgery, Digestive and Vascular Surgery Center, The First Affiliated Hospital, Xinjiang Medical University, Urumqi 830011, China.

[2]State Key Laboratory of Pathogenesis, Prevention and Treatment of High Incidence Diseases in Central Asia, Xinjiang Medical University, Urumqi 830011, China

[3]Department of Cardiac Surgery, The First Affiliated Hospital, Xinjiang Medical University, Urumqi 830011, China.

[4]Department of Critical Care Medicine, The First Affiliated Hospital, Xinjiang Medical University, Urumqi 830011, China.

[5]Department of Anesthesia, The First Affiliated Hospital, Xinjiang Medical University, Urumqi 830011, China.

[†]These authors contributed equally to this work.

[*]Correspondence

Tuerganaili Aji (tuergan78@sina.com) and Yingmei Shao (syingmei1@163.com), Department of Hepatobiliary and Echinococcosis Surgery, Digestive and Vascular Surgery Center, The First Affiliated Hospital, Xinjiang Medical University, Urumqi, China.

Supplementary tables

Table 1. Selected results from the initial evaluation following the patient's admission.

Variables	Result	Reference range
**Routine blood analysis**		
WBC (10^9/L)	5.28	3.50-9.50
NE (%)	78.4↑	40-75
LYMPH (10^9/L)	0.74↓	1.10-3.20
HGB (g/L)	141.00	115-150
PLT (10^9/L)	253.00	125-350
**Urinalysis**		
UPC	+↑	-
UWBC	+++↑	-
Urine color	Deep yellow	Light yellow
UWBCC (/μL)	95↑	0-28
**Fecal analysis**		
Occult blood	-	-
Fat	-	-
Worm eggs (/HPF)	-	-
**Coagulation profile**		
PT (S)	14.6	9.0-15.0
APTT (S)	32.5	23-38
INR	1.32↑	0.8-1.3
FBG (g/L)	3.76	2-4
**Liver function**		
TB (μmol/L)	31.56↑	3-22
DBIL (μmol/L)	0.30	0-5
IBIL (μmol/L)	26.66↑	0-19
ALB (g/L)	39.30	35-50
AST (U/L)	32.61	14-36
ALT (U/L)	18.00	9-52
GLU (mmol/L)	7.30↑	3.9-6.1
**Kidney function**		
BUN (mmol/L)	5.54	2.5-6.1
CREA (μmol/L)	54.47	46-92
**Electrolytes**		
K (mmol/L)	2.73↓	3.5-5.1
Na (mmol/L)	138.58	137-145
Cl (mmol/L)	100.9	98-107
Ca (mmol/L)	2.14	2.1-2.55
Mg (mmol/L)	0.79	0.7-1.0
P (mmol/L)	1.04	0.81-1.45
**Infection indicators**		
HBcAb (U/mL)	1.56↑	<0.7

**Tumor markers**			
AFP (ng/mL)	1.24	0-7	
CA19-9 (U/mL)	5.05	0-37	
**Cardiac function**			
FS (%)	33.33	25-45	
EF (%)	61.90	50-70	
SV (mL)	66.56	50-80	
CO (L/min)	5.86	4.00-6.00	
**Pulmonary function**			
Parameter	Tested	Estimated	%Estimated
VC (L)	2.32	2.91	79.7
ERV (L)	1.01		
IRV (L)	0.74		
TV (L)	0.57		
FVC (L)	2.19	2.91	75.3
FEV1.0 (L)	1.61	2.37	67.9
PEF (L/S)	4.22	5.63	75.0
MV (L/min)	49.3	92.6	53.2

Fasting blood samples were collected upon admission, with random timing for urine and stool samples. Cardiac function was evaluated using ultrasonic cardiogram (UCG). Parameters above the normal range are highlighted in red, while those below the normal range are indicated in blue. AFP, alpha-fetoprotein; ALB, albumin; ALT, alanine aminotransferase; APTT, activated partial thromboplastin time; AST, aspartate aminotransferase; BUN, urea nitrogen; Ca, calcium; CA19-9, carbohydrate antigen; Cl, chlorine; CO, cardiac output; CREA, creatine; DBIL, direct bilirubin; EF, (left ventricular) ejection fractions; ERV, expiratory reserve volume; FBG, fibrinogen; FEV, forced expiratory volume; FS, (left ventricular) fractional shortening; FVC, forced vital capacity; GLU, glucose; HBcAb, Hepatitis B core antibody; HGB, hemoglobin; HPF, high power field; IBIL, indirect bilirubin; INR, international normalized ratio; IRV, inspiratory reserve volume; K, kalium; L, litter; LYMPH, lymphocytes; Mg, magnesium; MV, minute volume; Na, sodium; NE, neutrophils; P, phosphorus; PEF, peak expiratory flow; PEI, PLT, platelets; PT, prothrombin time; S, second; SV, stroke volume; TB, total bilirubin; TV, tidal volume; UPC, urine protein; UWBC, urine white blood cell; UWBCC, urine white blood cell count; VC, vital capacity; WBC, white blood cells.

Table 2. Timeline of significant events, test results, diagnoses, and treatments after admission.	
**Time (POD)**	**Events and symptoms**, **test results and diagnosis**, **consultation**, **or main treatments (interventions)**
-33	Admission<br>Blood test: Jaundice, blood glucose↑, HBcAb↑, and hypokalemia<br>Urinalysis: Proteinuria (+) and potential urinary tract infection<br>Dynamic EKG: Sinus rhythm, occasional atrial premature beats (27 times in 24 hrs), ST-T changes<br>UCG: CAE (ambiguous location) and aortosclerosis<br>MRI: HAE, chronic cholecystitis, multiple right kidney cysts, and scattered lacunar cerebral infarction<br>Oral and intravenous potassium supplementation, and other symptomatic treatments
-32	ABPM: Average BP 109/69 mmHg, diastolic pressure load value higher than the normal range<br>Abdominal ultrasound: HAE and chronic cholecystitis<br>CTA: HAE, CAE, PAE, liver morphosis, splenomegaly, renal cysts, pulmonary atelectasis, slightly widened right branch of PV, disordered second porta hepatis, and possibly invaded, compressed, and narrowed post-hepatic IVC
-31	ULA: HAE<br>Dpt. Cardiac Surgery: Completion of ECG, chest CT, cardiac MRI, coronary CTA, and MDT discussion are suggested
-30	EKG: Sinus rhythm<br>CMRI: CAE
-29	UCA: CAE (ambiguous location) with IVC compression and diaphragm invasion<br>ABGA: Type I respiratory failure<br>Pulmonary function: Moderate mixed ventilation dysfunction and increased peripheral elastic resistance
-28	Dpt. Respiratory Medicine:<br>1. Doxofylline 0.3 g (ivgtt, qd)<br>2. Ipratropium bromide 2.0 ml (inh, bid)<br>3. Beclomethasone propionate 0.8 mg (inh, bid)<br>4. Montelukast sodium tablets 10 mg (po, hs)<br>5. Ambroxol hydrochloride injection 30 mg (ivgtt, bid)<br>6. Persistent low flow oxygen inhalation (1-3 L/min, longer than 15 hrs per day)<br>Dpt. Cardiology: No contraindications for surgery are reported
-27	UCG: CAE (ambiguous location)<br>MDT consensus:<br>1. Surgical rection of HAE and CAE lesions<br>2. Oral albendazole for PAE
-23	UCG: CAE (ambiguous location), aortosclerosis, mild mitral

	valve insufficiency, and mildly elevated pulmonary artery pressure
-22	Dpt. Respiratory Medicine:<br>1. Absolute surgical contraindications are ruled out<br>2. Continue improving lung function according to the previous prescriptions<br>Dpt. Psychiatry: No surgical contraindications are noted
-5	Pulmonary and abdominal CT(A): HAE, CAE, PAE, liver morphosis, splenomegaly, renal cysts, pulmonary atelectasis
-1	TEE: Right intra-atrial CAE<br>Preoperative MDT assessment:<br>1. Dpt. Anesthesiology:<br>(1) High anesthetic risk<br>(2) Close monitoring of bleeding and coagulation<br>(3) Extracorporeal circulation backup required<br>2. ICU:<br>(1) Extremely high perioperative risk<br>(2) Patient should be transferred to ICU after surgery<br>3. CT Room: Confirmed diagnosis of HAE, CAE, and PAE<br>4. Dpt. Cardiac Surgery: Solid mass present at RA and IVC entrance<br>5. Consensus:<br>(1) Surgical removal of HAE and CAE lesions<br>(2) Oral albendazole for PAE<br>Preoperative blood preparation:<br>1. RBC suspension 10 U<br>2. FFP 2000 mL<br>3. CRYO 10 U
0	Fasting<br>Surgical procedures:<br>1. Name:<br>(1) ELRA<br>(2) RA reconstruction<br>(3) Pericardial and diaphragmatic repair<br>2. Duration:<br>(1) Total: 17 hrs 40 mins<br>(2) RA repair: 25 mins<br>(3) IVC reconstruction: 49 mins<br>(4) Anhepatic phase: 3 hrs 45 mins<br>(5) Autotransplantation (Time consumed on graft anastomoses): 2 hrs 27 mins<br>3. Extracorporeal circulation:<br>(1) Total time: 1 hr 14 mins<br>(2) Heart-lung machine: Jostra H2O<br>(3) Extracorporeal membrane oxygenator (ECMO): TERUMO

CX*RX25RW

(4) Fluids: 1) RBC suspension: 4 U 2) Plasma substitute: 500 mL 3) Albumin: 100 mL 4) 0.9% Sodium chloride: 2000 mL 5) Remaining blood: 1600 mL 6) Total volume of preloading fluid: 3100 mL

(5) Urine: 1) Pre-perfusion: 400 mL; 2) During perfusion: 1300 mL

(6) Blood ultrafiltration: 1000 mL

(7) Coagulation adjustment: 1) Heparin: 250 mg; 2) Neutralization: Fish protamine 300 mg

(8) ACT: 1) Baseline 165 s; 2) Post-heparinization: 999 s; 3) In-process: 824 s; 4) Post-neutralization: 126 s

(9) Drugs used: 1) 25% Magnesium sulfate: 10 mL; 2) 10% Calcium chloride: 5 mL; 3) Furosemide: 20 mg; 4) 5% Sodium bicarbonate: 100 mL

4. Intravenous infusions:

(1) Cefuroxime sodium injection 4.5 g (ivgtt)

(2) 15% Potassium chloride (pi, 0.5-1.5 g/hr)

(3) Lycium barbarum acid sufentanil injection 850 μg (iv)

(4) Tartaric acid epinephrine injection (pi, 0.1-0.2 μg/kg·min)

(5) 10% Calcium gluconate injection (pi, 0.5-2.0 g/hr)

(6) Dexamethasone sodium phosphate injection 30 mg (ivgtt)

(7) Dopamine hydrochloride injection (pi, 3.0 μg/kg·min)

(8) Isoproterenol hydrochloride injection (pi, 50 mg/hr)

(9) Heparin sodium injection 250 mg (iv)

(10) Lidocaine hydrochloride 0.3 g (iv)

(11) Propofol medium/long chain fat emulsion injection (iv, 20 mg; pi, 240 mg/hr)

(12) Midazolam 2 mg (iv)

(13) Rocuronium bromide injection (iv, 50 mg; pi, 10 μg/kg·min)

(14) Nitroglycerin injection (pi, 0.2 μg/kg·min)

(15) Dexmedetomidine hydrochloride injection (pi, 0.2 μg/kg·min)

(16) Remifentanil hydrochloride injection (pi, 600 μg/hr)

(17) Human albumin injection 150 g (ivgtt)

(18) 0.9% Sodium chloride injection 2250 mL (ivgtt)

(19) Sodium acetate Ringer's injection 7000 mL (ivgtt)

(20) Sodium bicarbonate injection 180 mL (ivgtt)

(21) Hydroxyethyl starch 13004 electrolyte injection 500 mL (ivgtt)

(22) 5% Glucose injection 100 mL (ivgtt)

(23) Plasma 1430 mL (ivgtt)

(24) RBC suspension 11 U (ivgtt)

(25) Autologous blood transfusion 1000 mL (ivgtt)

	5. Fluid loss:<br>(1) Urine 8400 mL<br>(2) Blood: 2800 mL<br>6. Drainage tubes:<br>(1) Lesser omental foramen<br>(2) Subdiaphragmatic<br>(3) Pelvic<br>(4) Hepatic wound surface<br>(5) Pericardial and mediastinal<br>(6) Closed thoracic drainage<br>Patient transferred to ICU
1	APACHE score[1]: 14<br>OS: 100%<br>POCU: PHH<br>Respiratory support: Tracheal intubation with ventilator-assisted breathing<br>Through sputum suction via BAL<br>Long-term systemic treatment:<br>1. Monitoring of body temperature, blood glucose, blood pressure, pulse oximetry, and pulmonary function<br>2. Record 24-hour fluid intake and output<br>3. Anti-fever: Physical cooling<br>4. Oxygen inhalation<br>5. Mechanical-assisted sputum clearance<br>6. Anti-thrombotic: Limb pneumatic therapy<br>7. Anti-biotic: Imipenem and cilastatin sodium injection 1.0g (ivgtt, q8h)<br>8. Sputum suction
2	UCG: Aortosclerosis, mild mitral valve insufficiency, mildly elevated pulmonary artery pressure, and small amount of pericardial effusion<br>POCU: PHH<br>Pathology: HAE and CAE<br>Nutritional support: Retain gastric tube and nasogastric feeding of EN emulsion (500 mL, qd)<br>Sudden onset of AF at night: Peak HR 202 beats/min<br>Anti-AF:<br>1. Amiodarone hydrochloride injection 300 mg (pi, 10 mL/hr)<br>2. CED via intraoperatively implanted pacing lead on the surface of the patient's right ventricle<br>3. Esmolol hydrochloride injection 1 g (pi, 5 mL/hr)
3	Cardioprotective: Recombinant human brain natriuretic peptide injection 0.5 mg (ivgtt, q12h)
4	Ventilator-assisted breathing discontinued, endotracheal tube

	removed and high-flow nasal cannula oxygenation was given<br>POCU: PHH<br>Fluids:<br>1. 10% Glucose injection 500 mL+15% potassium chloride 10 mL+ human insulin injection 10 IU (ivgtt, qd)<br>2. 0.9% Sodium chloride injection 400 mL+10% sodium chloride injection 100 mL (ivgtt, qd)<br>Anti-biotic: Vancomycin hydrochloride injection 1000 mg (ivgtt, q12h)<br>Anti-thrombotic: Enoxaparin sodium injection 40 mg (sc, qd)<br>Hepatobiliary protective: Ursodeoxycholic acid 250 mg (ng, q12h)
5	Deteriorated liver function: TBIL 174.84 μmol/L, DBIL 73.78 μmol/L, IBIL 60.67 μmol/L<br>POCU: PHH<br>Dpt. Hepatology:<br>1. Adenosine disodium triphosphate injection 1 g (ivgtt, qd)<br>2. Compound glycyrrhizin injection 80 mg (ivgtt, qd)<br>3. Control infection, maintain stable circulation, stabilize electrolyte levels, and closely monitor liver function and infection markers
6	Ascitic bacterial culture: Negative<br>POCU: PHH and segmentally thin of post-hepatic IVC
7	Cough and phlegm<br>Bronchially collected sputum culture: *Pseudomonas aeruginosa*<br>UCG: Aortosclerosis and mild mitral valve insufficiency<br>POCU: PHH and segmentally thin of the post-hepatic IVC<br>ABGA: Mild respiratory alkalosis<br>Through sputum suction via BAL<br>Dpt. Acupuncture and Massage: Administer acupuncture, massage, medicinal cupping, and moxibustion to promote blood circulation, remove blood stasis, dredge meridians, and relieve pain (twice daily, for 10 days)<br>Liquid diet<br>Anti-biotic: Colistimethate Sodium injection 150 mg (q12h)<br>Cough suppressant and expectorant treatment:<br>1. Budesonide Suspension for Inhalation 1 mg+ ipratropium bromide solution for inhalation 0.5 mg (inh, q8h)<br>2. Doxofylline injection 0.2 g (pi, 10 mL/hr)<br>3. Ambroxol hydrochloride injection 30 mg (ivgtt, q12h)
8	ABGA: Mild respiratory alkalosis<br>Through sputum suction via BAL<br>POCU: PHH and segmentally thin of the post-hepatic IVC
9	Circulatory instability<br>ABGA: Mild respiratory alkalosis

	Through sputum suction via BAL<br>PICCO: Puncture site: left femoral artery
10	Dyspnea<br>ABGA: Mild respiratory alkalosis<br>POCU: PHH<br>CT: 1. PAE, inflammation in the lower right lung, partial consolidation in both lower lungs 2. Pericardial and right pleural effusion 3. Multiple calcified lymph nodes in mediastinum and both pulmonary hilus 4. PHH, splenomegaly, and accessory spleen<br>Through sputum suction via BAL<br>Nutritional support: Fat emulsion, amino acids (17) and glucose (11%) injection 1440 mL (ivgtt, qd)<br>Acid suppression: Omeprazole sodium injection 40 mg (ivgtt, q12h)<br>Cardioprotective: Ivabradine hydrochloride tablets 5 mg (ng, qd)<br>Endotracheal intubation-assisted breathing<br>CVC: Right subclavian vein<br>Plasma transfusion due to coagulation dysfunction: FVIP 240 mL
11	Critical condition, deteriorated respiratory function<br>Through sputum suction via BAL<br>Tracheotomy<br>Anti-biotic: Vancomycin hydrochloride injection 1000 mg (ivgtt, q12h)<br>Patient status: Deep sedation
12	POCU: PHH<br>Through sputum suction via BAL<br>Thoracic drainage tube removed<br>Maintaining BP:<br>1. Norepinephrine 5 mg (pi)<br>2. Dopamine 50 mg (pi, 5 mL/hr)
13	Severe rhonchi and unable to expectorate independently: Nebulized expectorant treatment continued<br>Intermittent prone positioning to prevent aspirational pneumonia
14	Pleural effusion culture: *Candida albicans*<br>Through sputum suction via BAL<br>MDR bacterial infection<br>Anti-biotic: Fluconazole sodium chloride injection 0.4 g (ivgtt, qd)
15	POCU: PHH<br>Anti-thrombotic: Enoxaparin sodium injection 40mg (sc, qd)<br>Pericardial and mediastinal drainage tube removed
16	Patient status: Conscious<br>Failed trial off ventilator<br>Blood culture: Negative
17	POCU: PHH

	Through sputum suction via BAL
19	Ultrasound: Bilateral pleural effusion<br>CT: 1. PAE, deteriorated inflammation in the lower right lung and minimal inflammation in the lingual segment of the upper left lobe 2. Unaltered pericardial effusion and increased pleural effusion combined with discoid atelectasis 3. Multiple calcified lymph nodes in mediastinum and both pulmonary hilus 4. PHH, splenomegaly and accessory spleen, and left adrenal hyperplasia<br>Right-sided thoracentesis drainage with pleural fluid bacterial culture<br>Lesser omental foramen, subdiaphragmatic, pelvic, hepatic wound surface, and pericardial and mediastinal drainage tubes removed
20	Through sputum suction via BAL<br>Hepatobiliary protective: Ursodeoxycholic acid 250 mg (ng, q12h)
21	Oxygen mask at the tracheostomy site<br>Normal BT, stable HR, OS, breathing, and BP<br>Patient transferred back to general ward<br>Cough suppressant and expectorant treatment:<br>(1) Budesonide suspension for Inhalation 1 mg (inh, bid)<br>(2) Ipratropium bromide solution for inhalation 0.5 mg (inh, bid)<br>(3) Doxofylline injection 0.3 g (ivgtt, qd)<br>(4) Ambroxol hydrochloride injection 30 mg (ivgtt, bid)<br>Acid suppression: Omeprazole sodium injection 40 mg (ivgtt, q12h)<br>Analgesic: Flurbiprofen axetil injection 50 mg (ivgtt, q12h)<br>Hepatobiliary protective: Ursodeoxycholic acid 250 mg (ng, q12h)<br>Anti-biotic:<br>(1) Fluconazole sodium chloride injection 0.4 g (ivgtt, qd)<br>(2) Imipenem and cilastatin sodium injection 1.0g (ivgtt, q8h)<br>(3) Vancomycin hydrochloride injection 1.0g (q12h)
22	Tachycardia, and gastrointestinal dysfunction<br>POCU: PHH<br>Dpt. Nutrition: EN 500mL (po, qd); PN 800mL (pi, 40 mL/hr, qd)<br>Dpt. Respiratory Medicine: The patient is presently on a regimen of imipenem, fluconazole, and vancomycin for infection management. Continue sputum drainage, along with repeated comprehensive sputum microbiological culture and sensitivity testing. Regular monitoring of liver and renal function is also crucial<br>Dpt. Acupuncture and Massage: Acupuncture and massage therapy should be administered for a minimum of 2 treatment courses, with 1-2 sessions per day<br>HR lowering drug: Bisoprolol fumarate tablets 5 mg (po, qd)<br>Anti-thrombotic: Rivaroxaban tablets 10 mg (po, qd)

	Anti-biotic: Fluconazole sodium chloride injection discontinued<br>Midline catheter implanted in vena basilica by PICC treatment unit
23	Deteriorated renal function<br>CT: 1. PAE, improved inflammation in the lower right lung 2. Basically absorbed pleural effusion 3. Multiple calcified lymph nodes in mediastinum and both pulmonary hilus 4. Decreased PHH, splenomegaly and accessory spleen, and left adrenal hyperplasia<br>Dpt. Nephrology: AKI due to vancomycin-induced nephrotoxicity is considered and prescription of following renal protective drugs is recommended<br>1. Compound α-ketoacid tablets 4 tablets (po, tid)<br>2. Shenwu Yishen tablets 4 tablets (po, tid)<br>3. Sodium bicarbonate tablets 2 tablets (po, tid)<br>4. Furosemide injection 20 mg (iv, bid)<br>5. Spironolactone tablets 20 mg (po, qd)<br>6. Close monitoring of renal function and electrolyte<br>7. Avoid using nephrotoxic drugs<br>8. Hemodialysis should be considered if necessary<br>Dpt. Cardiology: Suspicious heart failure due to infection, following interventions are recommended<br>1. Continuous monitoring of vital signs<br>2. Diuretic medication: Furosemide 20-40 mg (iv, bid) and spironolactone tablets 20 mg (po, qd)<br>3. Vasodilation: Nitroglycerin injection 30 mg (pi, 1 mL/hr)<br>4. Inotropic therapy: Deslanoside 0.2 mg (pi, qd) and digoxin tablets 0.125 mg (po, qd)<br>5. Monitor urine output, electrolytes, and changes in liver and kidney function
24	Bronchially collected sputum culture and antibiotic sensitivity testing: *Pseudomonas aeruginosa*, sensitive to cefoperazone-sulbactam<br>Vancomycin trough concentration: >100 μg/mL↑<br>Anti-biotic:<br>1. Vancomycin hydrochloride injection discontinued<br>2. Cefoperazone sodium and sulbactam sodium injection 3.0 g (ivgtt, q12h)
25	Anxiety, poor sleep quality, and insufficient sleep<br>Coughing with yellow and sticky sputum and difficult to expectorate independently<br>Bacterial culture of abdominal drainage fluid: Negative<br>Dpt. Psychiatry: Lorazepam 0.5-1.0 mg (po, hs)<br>Dpt. Respiratory Medicine: The patient has excessive sputum and consolidated changes in the lower right lung. Intensified airway

	management and humidification should be implemented. If necessary, sputum suction can be performed under bronchoscopy and sputum should be examined
26	Poor effective nebulized sputum suction<br>Thoracentesis drainage tube removed<br>Dpt. Rehabilitation: The bedside sputum suction and thromboprophylaxis rehabilitation guidance are provided and completion of D-dimer, BNP, and bilateral lower extremity and iliac vascular ultrasound is suggested<br>Dpt. Otolaryngology: Tracheostomy tube replacement and continuation of current nebulization treatment is recommended
27	Involuntary tremors below the right elbow, accompanied by numbness<br>UCG: Aortosclerosis and mild mitral valve insufficiency<br>Dpt. Nutrition: Adjust PN to 600 mL and continue current EN formula.
29	D-dimer: 3558 ng/mL↑<br>Pleural puncture fluid bacterial culture and antibiotic sensitivity testing: *Acinetobacter baumannii*, sensitive to tigecycline<br>Ultrasound: Right renal sinus separation, multiple cysts of the right kidney, and left calf intermuscular vein thrombosis<br>Anti-thrombotic: Enoxaparin sodium injection 40mg (sc, qd)<br>Dpt. Neurology: It is recommended to perform a comprehensive MRI with diffusion-weighted imaging of the head and skull to clarify the cause of the involuntary tremors and numbness below the right elbow
30	Blood-stained sputum and hypokalemia<br>Vancomycin trough concentration: 39.22 μg/mL↑<br>Dpt. Rehabilitation: Continue previous bedside sputum suction and thromboprophylaxis rehabilitation strategy<br>Dpt. Respiratory Medicine: It is advisable to perform a pulmonary ECT to rule out pulmonary embolism. Additionally, a thorough evaluation of the heart is recommended, including ECG, BNP, and cardiac biomarkers
31	CT: 1. PAE 2. Newly developed pleural effusion 3. Multiple calcified lymph nodes in mediastinum and both pulmonary hilus 4. Decreased PHH, splenomegaly and accessory spleen, and left adrenal hyperplasia<br>Nutritional support: Whole protein EN powder 250 mL (po, bid)<br>Dpt. Otolaryngology: Endotracheal intubation can be sealed
32	Tracheal tube replacement<br>Potassium supplement: Potassium chloride extended-release tablets 1 g (po, tid)
33	D-dimer: 2634 ng/mL↑

	CRE: 277.44 μmol/L↑
34	Vancomycin trough concentration: 22.17 μg/mL↑
35	UCG: Aortosclerosis and mild mitral valve insufficiency<br>ULA: PHH<br>Dpt. Cardiac Surgery: The temporary pacemaker lead for the right ventricle can be removed
36	Temporary pacemaker lead removed
37	Video laryngoscopy: Bilateral vocal cords are normal<br>Anti-biotics: All discontinued
38	Endotracheal tube removal and laryngotracheal fistula repair
41	UCG: Aortosclerosis and mild mitral valve insufficiency<br>CT: 1. PAE 2. Decreased pleural effusion 3. Multiple calcified lymph nodes in mediastinum and both pulmonary hilus 4. PHH, splenomegaly and accessory spleen, and left adrenal hyperplasia
42	Discharge<br>Instructions:<br>1. Analgesic: Dexketoprofen trometamol tablets 12.5 mg (po, prn)<br>2. Anti-thrombotic: Rivaroxaban tablets 20 mg (po, qd)<br>3. Diet and nutrition:<br>- Avoid spicy and irritating foods<br>-Stay hydrated<br>-Enhance nutrition with a balanced diet<br>4. Rehabilitation:<br>-Engage in moderate physical activity, avoid heavy physical labor<br>-Take one month of complete rest<br>5. PAE treatment:<br>-Start taking albendazole tablets once liver function is normal (po, 400 mg in the morning and 200 mg in the evening) for at least one year<br>-Liver function monitoring: Weekly in the first two weeks, biweekly in the following month, and then monthly. Temporarily cease albendazole if liver function is abnormal, and resume until it returns to normal.<br>6. Remove tracheostomy sutures one week after discharge<br>7. Follow-up:<br>-OPD follow-up one month after discharge<br>-Regular follow-up visits in every three month after discharge and complete routine blood tests, abdominal ultrasound, and chest CT scan in every visit. Abdominal CT scan is advised in every six months<br>8. Seek medical attention promptly if any discomfort is experienced

ABGA, arterial blood gas analysis; ABPM, ambulatory blood pressure monitoring; ACT: activated coagulation time bid, bis in die; AF, atrial fibrillation; AKI, acute kidney injury; APACHE, acute physiology and chronic health evaluation; BAL, bronchoscopic alveolar lavage; BNP, brain natriuretic peptide; BP, blood pressure; BT, body temperature; CAE, cardiac alveolar echinococcosis; CED, cardiac electrical defibrillation; (C)MRI, (cardiac) magnetic resonance imaging; CRE, creatine; CRYO, cryoprecipitate coagulation factor; CT(A), computed tomography (angiography); CVC, central venous catheterization; DBIL, direct bilirubin; Dpt., department; ECT, emission computed tomography; EKG, electrocardiogram; ELRA, *ex vivo* liver resection and autotransplantation; EN, enteral nutrition; FFP, fresh frozen plasma; FVIP, fresh virus-inactivated plasma; HAE, hepatic alveolar echinococcosis; HBcAb, hepatitis B core antibody; HR, heart rate; hr, hour; hs, hora somni; HV, hepatic vein; IBIL, indirect bilirubin; ICU, intensive care unit; inh, inhalation; IVC, inferior vena cava; iv, intravenous; ivgtt, intravenous drip; MDR, multi-drug resistant; MDT, multi-disciplinary treatment; min, minute; ng, nasalgastric; OPD, outpatient department; OS, oxygen saturation; PAE, pulmonary alveolar echinococcosis; PHH, perihepatic hematoma; pi, pump injection; PICC, peripherally inserted central venous catheterization; PICCO, pulse indicator continuous cardiac output; PN, parenteral nutrition; po, peros; POCU, point-of-care ultrasound; POD, postoperative day; prn, pro re nata; qd, quaque die; RA, right atrium; RBC, red blood cell; sc, subcutaneous; TBIL, total bilirubin; TEE, transesophageal echocardiography; TNA, total nutrition admixture; UCA, ultrasonic cardiac angiography; UCG, ultrasonic cardiogram; ULA, ultrasonic liver angiography.

Supplementary figures

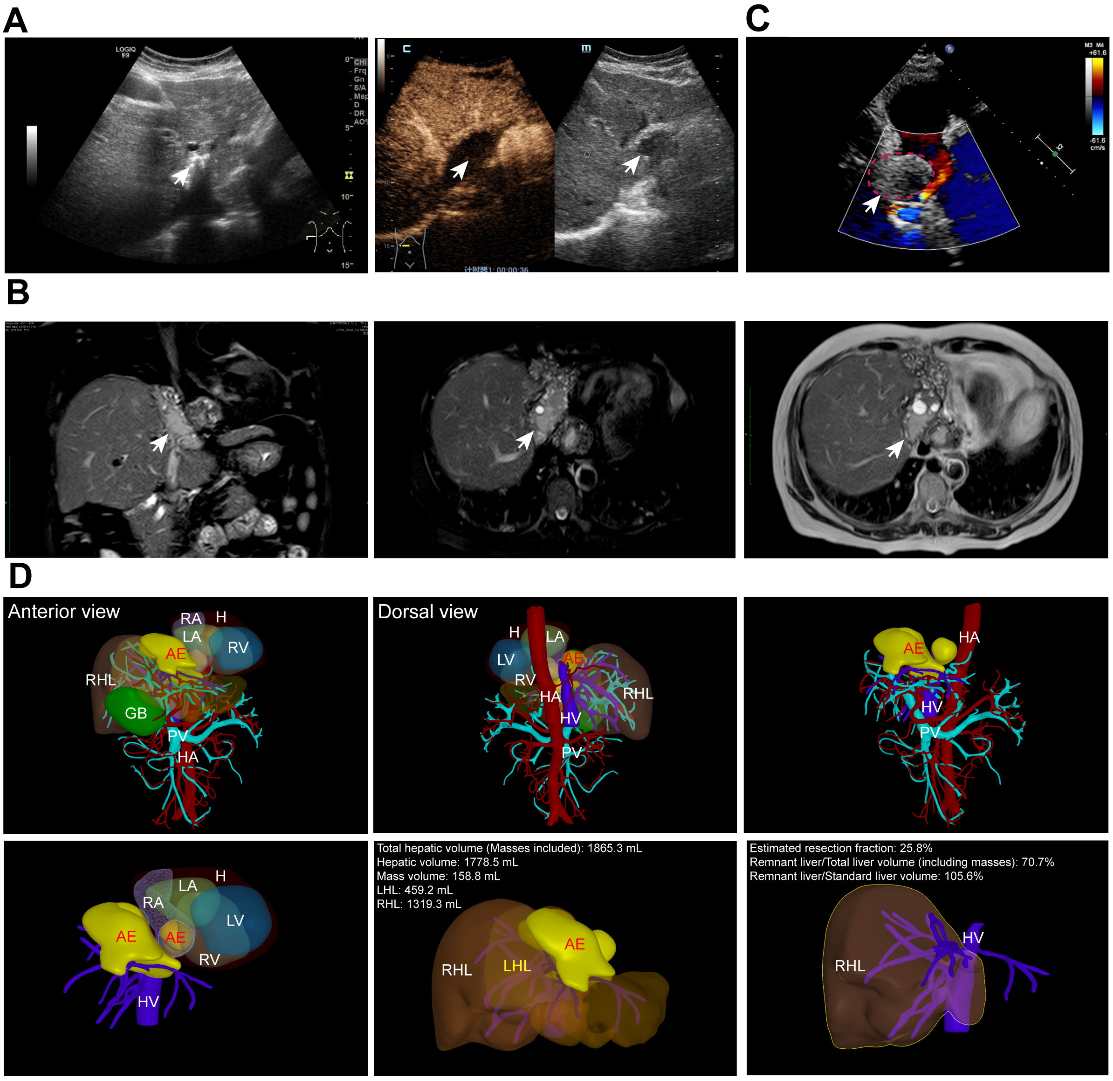

Fig. 1 Preoperative imaging and liver volumetry of the patient with AE masses. (A) The abdominal ultrasound (left) and ultrasonic liver angiography (ULA, right), revealing morphological abnormalities and enlargement in the liver post-hepatic alveolar echinococcosis surgery. An irregular medium-to-high echo lesion (approximately 8.0x3.0x6.2 cm) is observed at the liver's left-right lobe junction, characterized by unclear boundaries, uneven internal echoes, and diffuse calcified dot-like strong echoes. The lesion, indicated by white arrows, exhibits a rim-like high enhancement with no apparent internal enhancement and a central irregular anechoic area with poor sound transmission. This lesion encircles and adheres to blood vessels, compressing the bile duct (inner diameter: 0.6 cm). **(B)** Different phases of the MRI scan, displaying irregular mixed signal lesions (indicated by white arrows) in the patient's liver's left lobe, caudate lobe, and tail lobe. The lesion's boundary is unclear, measuring approximately 7.96x3.45 cm at its maximum range. The left branch of the PV is partially unclear, and the HVs are thickened. **(C)** An echoic mass (approximately 45x35 mm) at the RA and IVC entrance (marked by a white arrow and dashed red circle), devoid of blood flow signal. The IVC demonstrates compression and increased blood flow speed (1.25m/S). Echocardiography (ECG) also detected aortic sclerosis, mild mitral valve insufficiency, and mild pulmonary artery pressure elevation. **(D)** Patient's liver's three-dimensional (3D) reconstruction and volumetry (Available at:

https://yun.gradient3d.com/scene/case3d.html?ValidCode=230709&SID=b94e05f8-4e36-4773-8c3c-29fa31696896). AE, alveolar echinococcosis; GB, gallbladder; H, heart; HA, hepatic artery; HV, hepatic vein; LA, left atrium; LHL, left half liver; LV, left ventricle; PV, portal vein; RA, right atrium; RHL, right half liver; RV, right ventricle. Full images can be viewed in uploaded videos.

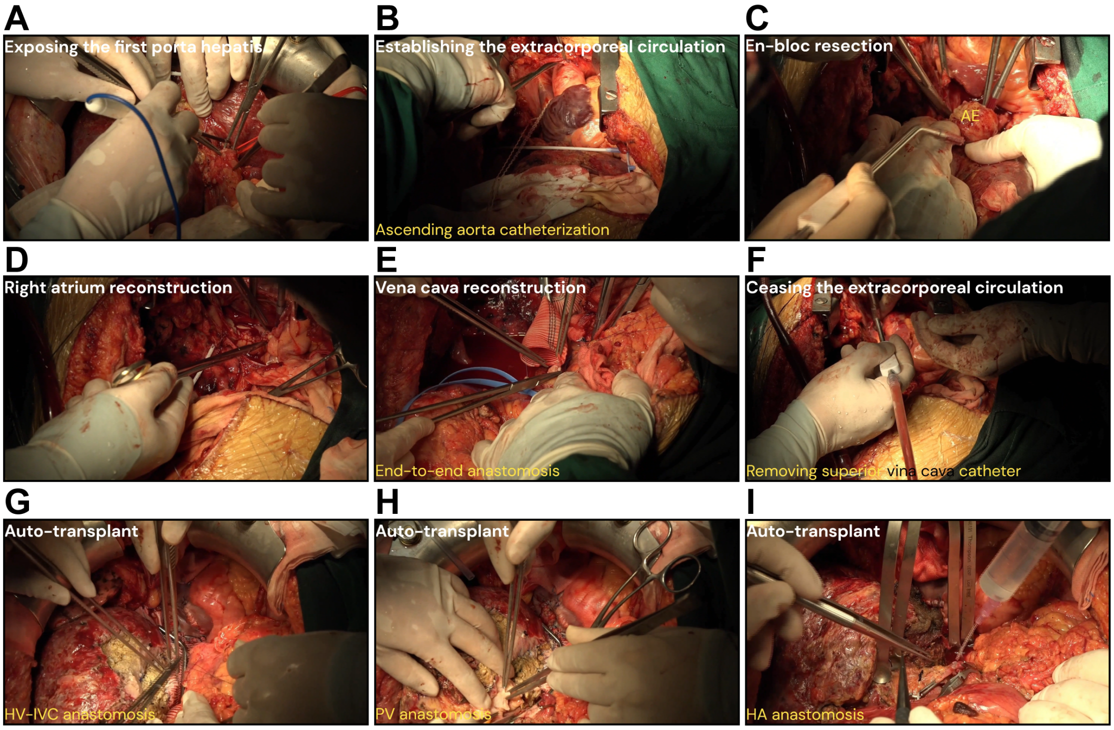

Fig. 2 The main steps of ELRA and RA reconstruction. (A-I) The fundamental steps of the surgical procedures. AE, alveolar echinococcosis; ELRA, *ex vivo* liver resection and Autotransplantation; HA, hepatic artery; HV, hepatic vain; IVC, inferior vena cava; PV, portal vein. For a thorough understanding of the surgical procedures, please refer to the accompanying videos.

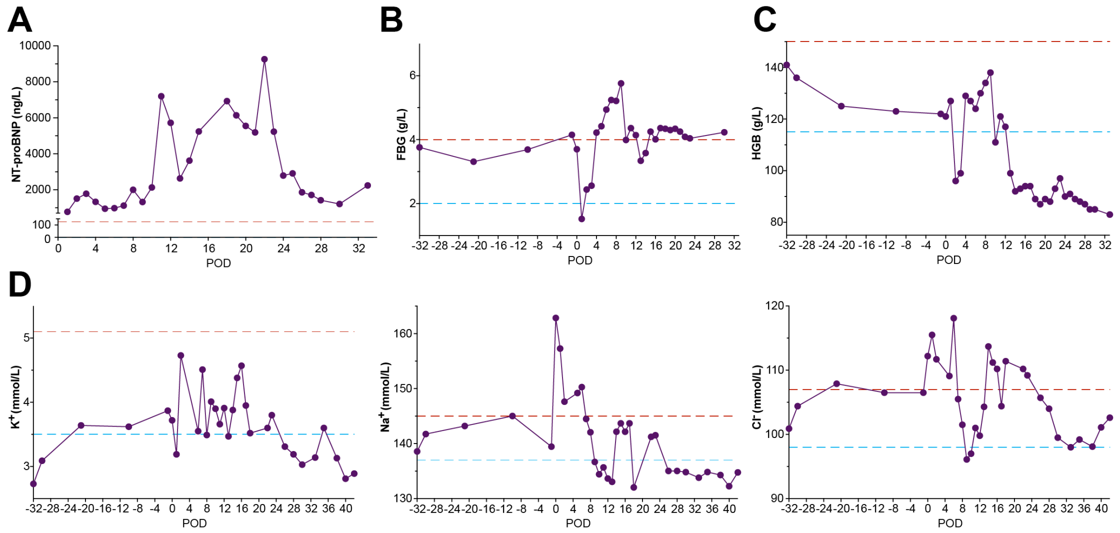

Fig. 3 Perioperative laboratory results of the patients. The upper and lower boundaries of normality are respectively denoted by dashed red and blue lines. FBG, fibrinogen; HGB, hemoglobin; NT-proBNP, N-terminal pro-B-type natriuretic peptide.

Supplementary video index

No.	File Name/Prefix	Content
1-1	1-1. POD-1-Transesophageal echocardiography (TEE)	Preoperative Transesophageal echocardiography
2-1	2-1. POD-31-CTA	CTA scans on POD-31
2-2	2-2-POD23-CT	CT scans on POD23
2-3	2-3-POD30-CT	CT scans on POD30
2-4	2-4-POD42-CT	CT scans on POD42
3-1	3-1. POD-32-MRI	MRI scans on POD-32
3-2	3-2. POD-30-Cardiac MRI	Cardiac MRI scan on POD-30
4-1	4-1.Surgical Procedures	Six-minute video of the whole surgical procedures
4-2	4-2.En-bloc resection	Separate short clip of *en bloc* resection

References

1. Goldhill DR, Sumner A. APACHE II, data accuracy and outcome prediction. *Anaesthesia*. 1998;53(10):937-943.
